# Giant fibroadenoma of the breast: A rare case in a mature woman

**DOI:** 10.1016/j.ijscr.2019.09.015

**Published:** 2019-09-20

**Authors:** Xiangyue Meng, Kosho Yamanouchi, Sayaka Kuba, Chika Sakimura, Michi Morita, Kunihito Matsuguma, Kengo Kanetaka, Mitsuhisa Takatsuki, Kuniko Abe, Susumu Eguchi

**Affiliations:** a Department of Surgery, Nagasaki University Graduate School of Biomedical Sciences, 1-7-1 Sakamoto, Nagasaki, 852-8501, Japan; b Department of Pathology, Nagasaki University Hospital, 1-7-1 Sakamoto, Nagasaki, 852-8501, Japan; c Department of Pathology, Nagasaki Genbaku (Atomic Bomb) Hospital, 3-15 Mori-machi, Nagasaki, 852-8511, Japan

**Keywords:** Giant fibroadenoma, Breast, Phyllodes

## Abstract

**Introduction:**

Fibroadenomas are common benign lesions of the breast that are usually found young patients. Giant fibroadenomas are uncommon benign lesions, defined as fibroadenomas of >5 cm in size, which are usually found in patients of less than 20 years of age.

**Presentation of case:**

A 39-year-old premenopausal woman presented with a right breast tumor that had rapidly increased in size and which showed ulceration and bleeding. Needle biopsy showed mixed connective tissue and an epithelial tumor without a leaf-like pattern, but indeterminate. Total mastectomy and skin grafting were performed. Histopathology confirmed the diagnosis of giant fibroadenoma.

**Discussion:**

In comparison to all previous reports on patients with giant fibroadenoma, this patient was relatively old and the etiology was unknown. Although rare, an appropriate therapeutic strategy should be decided according to the results of a histopathological examination.

**Conclusion:**

Early treatment could allow breast preserving surgery and patients should be recommended to undergo reexamination with awareness of progression.

## Highlights

• We experienced giant fibroadenoma of breast, a rare case in a mature woman.
• We suspected a malignant tumor, due to the size and ulceration and bleeding.
• Total mastectomy and skin grafting were needed for complete resection.
• An early diagnosis and treatment could prevent total mastectomy.

## 1 Introduction

Fibroadenomas in the breast are common benign lesions in women of less than 30 years of age. They feature polyclonal proliferation of both epithelial and stromal tissue, and are hyperplastic lesions rather than true neoplasms. Fibroadenomas usually present as hard, mobile, painless, obvious boundaries and easily accessible nodules [1]. Giant fibroadenomas, which are defined as fibroadenomas of greater than 5 cm in size, or 500 g, are rare benign breast lesions that account for approximately 0.5%–2% of fibroadenomas, and usually occur in women during pregnancy or lactation or in adolescent women [[2], [3], [4]]. We herein report the case of a 39-year-old woman with a giant fibroadenoma of >20 cm in diameter that was accompanied by ulceration and bleeding.

The work has been reported in line with the SCARE criteria [5].

## 2 Presentation of case

**Fig. 1:**
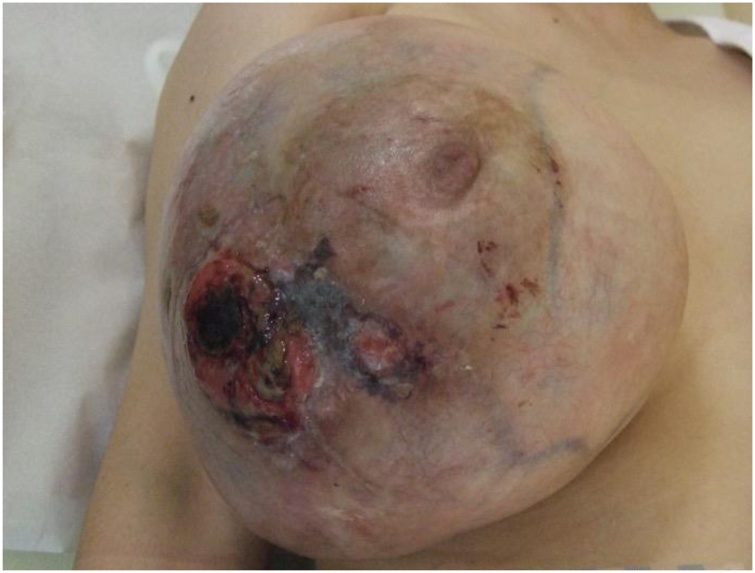
Large right breast mass with bleeding and ulceration on physical examination.

**Fig. 2:**
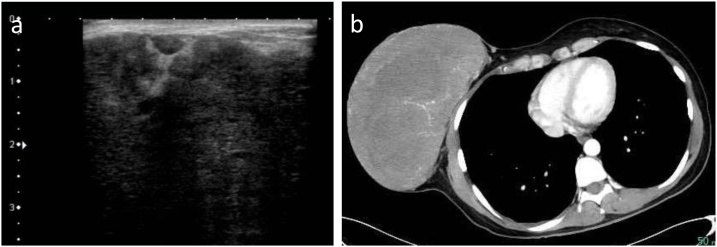
**a** Ultrasonography shows a circumscribed iso-echoic to hypo-echoic mass replacing the right breast tissue. **b** Enhanced computed tomography shows a huge tumor, with of 20 cm in diameter with poor internal enhancement.

**Fig. 3:**
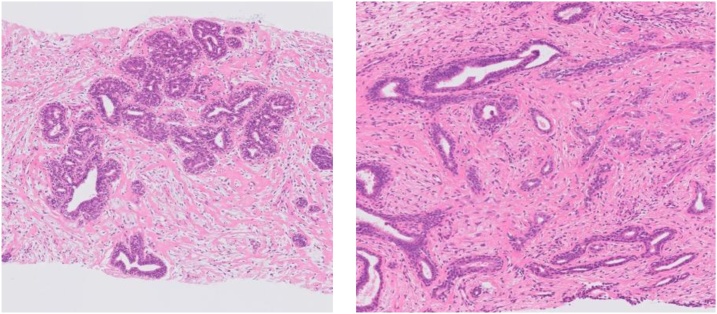
Core needle biopsy of the right breast mass shows mixed connective tissue and an epithelial tumor lined by a bilayered epithelium without leaf-like pattern (hematoxylin and eosin).

The patient was a 39-year-old woman who had become aware of a right breast mass 7 months previously. The mass had rapidly increased in size with the manifestation of ulceration and bleeding. She came to the hospital for emergency hemostasis. Her menstrual cycles were regular and she was not on any hormone therapy. A clinical examination showed a firm and mobile breast tumor on the outside of the right breast of approximately 20 cm in size, with a necrotic part in the lower quadrant of the breast, thickening of the surrounding skin, and capillary protrusion (Fig. 1). The right axillary lymph nodes were slightly swollen. She had a fever of >38 °C. Her serum level of carcinoembryonic antigen (CEA) was 15.4 ng/ml (reference range: <5 ng/ml). Ultrasonography (US) showed a relatively clear boundary of the iso- to hypo-echoic tumor, with partial multiple cysts (Fig. 2a). Computed tomography (CT) showed a huge tumor replacing the right breast tissue with a long diameter of approximately 20 cm, with poor internal enhancement, accompanied by areas of hypodensity, possibly due to degenerative necrosis (Fig. 2b). Based on these findings, a phyllodes tumor was suspected. CT did not show any evidence of distant metastasis. The right axillary lymph nodes were slightly swollen in comparison to the contralateral lymph nodes. A core needle biopsy revealed mixed connective tissue and an epithelial tumor without leaf-like pattern, with positivity for CD34, desmin (focal), and p63 (Fig. 3). Ki-67 was focally positive (less than 1% of the cells of the stroma). The probable diagnoses were borderline malignant phyllodes tumor and fibroadenoma, but were indeterminate. US-guided fine needle aspiration cytology of the right axillary lymph node showed no evidence of metastasis.

**Fig. 4:**
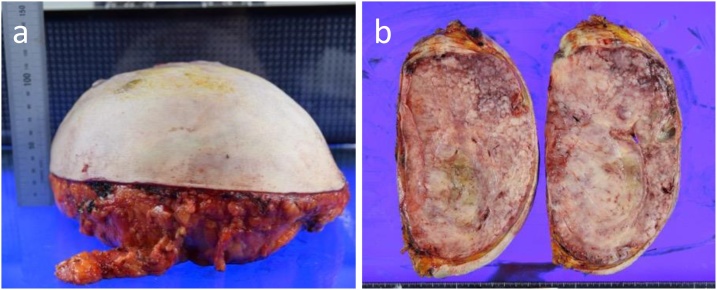
**a** The excised specimen. The specimen was 23.5 × 24.5 × 11 cm in size and weighed 3.2 kg. **b** The divided face of the tumor.

**Fig. 5:**
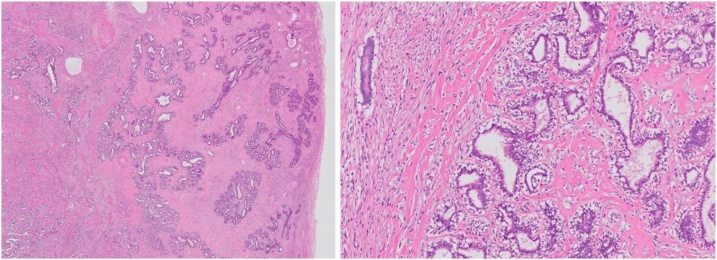
The surgical specimen shows fiboroadenoma, lined by a bilayered epithelium with a pericanalicular pattern (hematoxylin and eosin staining).

The patient underwent total mastectomy and skin grafting, with skin taken from the lower abdomen under general anesthesia. The actual size of the tumor was 23.5 × 24.5 × 11 cm and the weight was 3.2 kg (Fig. 4). The patient recovered well and her serum level of CEA dropped to the reference range after surgery. The boundary of the tumor section was consistent. A pathological examination was performed by an experienced pathologist, K. A., certified by the Japanese Society of Pathology and showed that the content of the tumor was almost consistent with the contents of multiple white nodules. Spindle cells proliferated in the stromal component with hyalinosis and the epithelial component was also hyperplastic. The glands were lined by a bilayered epithelium with a pericanalicular pattern of fibroadenoma and the absence of cytological atypia. The final histopathological diagnosis was giant fibroadenoma of the breast (Fig. 5).

## 3 Discussion

While fibroadenomas are common benign lesions in breast tissue, giant fibroadenomas are very rare. Giant fibroadenomas are usually encountered in pregnant or lactating women. Their growth is associated with increases in estrogen, progesterone, and prolactin [2]. Those found in adolescent woman (15–25 years of age) are referred to as juvenile giant fibroadenoma [[1], [2], [3]]. In our case, the patient was relatively old age in comparison to previously reported cases and was not lactating or pregnant. To our knowledge, this is the oldest giant fibroadenoma patient ever reported.

Initially, we suspected a malignant tumor, such as a malignant phyllodes tumor or breast cancer, due to the size and the increase in size over a short period with ulceration and bleeding. US is non-invasive modality and is useful in the diagnosis of lesions in breast tissue. On US, fibroadenomas appear as a well circumscribed round or oval solid mass, with weak internal echoes in a uniform distribution and intermediate acoustic attenuation [[2], [3], [4],^6^]. Smith et al. [7], however, reported that accuracy of US in the diagnosis of fibroadenoma was only 78.8% based on the evaluation of 447 patients of ≤25 years of age.

Both fibroadenomas and phyllodes tumors consist of proliferating epithelial and stromal cells. The differential diagnosis between fibroadenomas and phyllodes tumors is clinically important due to differences in therapeutic strategies. Fibroadenomas can be followed up safely or simply enucleated, while phyllodes tumors must be treated surgically [8]. Furthermore, phyllodes tumors must be resected with adequate margins (the most advocated surgical margin is 1 cm) to avoid local recurrence [9,10]. Reviews with large number of cases showed giant fibroadenomas can be usually completely excised with preservation of the developing breast parenchyma and nipple areolar complex, and mastectomy and breast reconstruction is uncommon but may be necessary in certain cases, such as ours [3,11]. Furthermore 20–30% of phyllodes tumors are malignant [8], and approximately 25% of malignant phyllodes tumors metastasize [12]. Phyllodes tumors occur in patients who are around 10–15 years older than those presenting with fibroadenomas [13]. Fibroadenomas are characterized by well demarcated margins, minimal atypia and rare mitosis while phyllodes tumors sometimes show invasive margins, matrix overgrowth and significant atypia and a leafy structure [14,15]. US shows a round or lobulated tumor, with enhanced echo and cystic intramural areas; phyllodes tumors are more frequently encountered than fibroadenomas [8,16]. However, it is often difficult to distinguish between these two entities because of the considerable overlap in their imaging features [17]. Because the examination of small samples from needle biopsy may yield uncertain results, the final diagnosis is sometimes made based on the pathological examination of the resected specimen. In the present case, the pathological diagnosis of giant fibroadenoma was made based on the examination of the surgical specimen.

In our case, the preoperative CEA level was mildly elevated and decreased to the normal range after surgery. We could not find any malignant features in the resected specimen. CEA is a glycoprotein involved in cell adhesion, which is normally produced in gastrointestinal tissue during fetal development [18]. The level of CEA in the blood of healthy adults is usually very low. However, the serum levels increase in some types of cancers, including breast cancer. CEA elevation was reported to be observed in at most 0.6% of patients with benign breast disease [19]. In a single institutional study, in the follow-up of patients with resected colorectal cancer, the false-positive elevation of CEA level was seen in 49.2% (358 out of 718 patients) of patients, with only 1.4% of them showing values greater than 15 ng/ml [20]. Other than cancer, the causes of CEA elevation include gastritis, peptic ulcer disease, diverticulitis, liver disease, chronic obstructive pulmonary disease, diabetes, and any acute or chronic inflammatory state [21]. In the present case, the patient had high fever without any other cause and the fever subsided after mastectomy. We hypothesized that the patient had been in an inflammatory state before surgery due to the huge tumor, which led to the elevation of the serum level of CEA.

## 4 Conclusion

Skin grafting or plastic surgery might be needed for patients who undergo mastectomy for giant fibroadenoma. An early diagnosis and treatment could prevent this situation and patients should be recommended to undergo reexamination with awareness of progression.

## Funding

No funding on this study.

## Ethical approval

Approval to publish this case report was waived by the institution.

## Consent

Written informed consent for publication of this case report and accompanying images was obtained from the patient. A copy of the written consent is available for review by the Editor-in-Chief of this journal, on request.

## Author’s contribution

KY, SK, CS, KM, and NH performed the surgery and were responsible for the care of the patient. KA performed the pathological examination. MX, KY, and CS designed and drafted the manuscript. MM, KK, MT, KA and SE reviewed and revised the manuscript.

## Registration of research studies

This is not systematic review or meta-analysis. Also this is not randomised clinical trial.

## Guarantor

Kosho Yamanouchi MD, PhD.

## Submission declaration

The authors declare that the work described has not been published previously, that it is not under consideration for publication elsewhere, that its publication has been approved by all authors and either tacitly or explicitly by the responsible authorities where the work was carried out, and that, if accepted, it will not be published elsewhere—including electronically in the same form in English or any other language—without the written consent of the copyright holder.

## Provenance and peer review

Not commissioned externally peer reviewed.

## Declaration of Competing Interest

The authors report no conflict of interest.
